# Cytokine Disturbances in Coronary Artery Ectasia Do Not Support Atherosclerosis Pathogenesis

**DOI:** 10.3390/ijms19010260

**Published:** 2018-01-16

**Authors:** Usama Boles, Anders Johansson, Urban Wiklund, Zain Sharif, Santhosh David, Siobhan McGrory, Michael Y. Henein

**Affiliations:** 1 Department of Public Health and Clinical Medicine, Umeå University, 901 87 Umeå, Sweden; bolesu@tcd.ie (U.B.); anders.p.johansson@umu.se (A.J.); 2 Cardiology Department, Letterkenny University Hospital, Letterkenny, F92 AE81, Co. Donegal, Ireland; zainsharif@rcsi.ie (Z.S.); santhosh.tharakan@gmail.com (S.D.); siobhanmcgrory@yahoo.co.uk (S.M.); 3 Molecular Periodontology and Odontology, Umeå University, 901 87 Umeå, Sweden; 4 Department of Radiation Sciences, Umeå University, 901 87 Umeå, Sweden; urban.wiklund@umu.se; 5 Molecular & Clinical Sciences Research Institute, St. George University, London SW17 0RE, UK

**Keywords:** coronary artery ectasia, atherosclerosis, cytokines, macrophage activation, immune inflammatory response, coronary artery disease

## Abstract

Background: Coronary artery ectasia (CAE) is a rare disorder commonly associated with additional features of atherosclerosis. In the present study, we aimed to examine the systemic immune-inflammatory response that might associate CAE. Methods: Plasma samples were obtained from 16 patients with coronary artery ectasia (mean age 64.9 ± 7.3 years, 6 female), 69 patients with coronary artery disease (CAD) and angiographic evidence for atherosclerosis (age 64.5 ± 8.7 years, 41 female), and 140 controls (mean age 58.6 ± 4.1 years, 40 female) with normal coronary arteries. Samples were analyzed at Umeå University Biochemistry Laboratory, Sweden, using the V-PLEX Pro-Inflammatory Panel 1 (human) Kit. Statistically significant differences (*p* < 0.05) between patient groups and controls were determined using Mann–Whitney *U*-tests. Results: The CAE patients had significantly higher plasma levels of INF-γ, TNF-α, IL-1β, and IL-8 (*p* = 0.007, 0.01, 0.001, and 0.002, respectively), and lower levels of IL-2 and IL-4 (*p* < 0.001 for both) compared to CAD patients and controls. The plasma levels of IL-10, IL-12p, and IL-13 were not different between the three groups. None of these markers could differentiate between patients with pure (*n* = 6) and mixed with minimal atherosclerosis (*n* = 10) CAE. Conclusions: These results indicate an enhanced systemic pro-inflammatory response in CAE. The profile of this response indicates activation of macrophages through a pathway and trigger different from those of atherosclerosis immune inflammatory response.

## 1. Introduction

Coronary artery ectasia (CAE) is seen in 1.5–5% of patients undergoing coronary angiography, with predominance in males. It is defined as dilatation of an arterial segment to a diameter at least 1.5 times that of an adjacent normal artery and involves at least one third of the affected artery [1,2].

The exact pathogenesis of CAE is not well established [3]. It is attributed to atherosclerosis in 50% of cases based on its common association [4], but recent extended lipidomic profiling has provided evidence against this [5]. Up to 20% of CAE cases are considered congenital, and 10–20% are associated with inflammatory or connective tissue diseases, e.g., Ehlers–Danlos syndrome, Kawasaki disease, and polycystic kidney disease [6,7]. Iatrogenic coronary aneurysms after balloon angioplasty [8,9] and Takayasu arteritis (TA) [10] represent rare causes for ectasia.

Atherosclerosis is a chronic process [11] triggered by an immuno-inflammatory reaction as a response to endothelial injury [12,13] and hence enhances thrombus formation [14]. Triggered cytokine reactions remain the mainstay of progressive atherosclerosis mechanisms [15]. Principally, activated macrophages initiate the innate immune response that secretes tumor necrosis factor (TNF-α), which eventually promotes atherogenesis [16]. On the other hand, high levels of IL-10 [17,18,19] and low levels of IL-4 [20] and IL-8 are protective against atherosclerosis through suppressing macrophages activity [21,22].

In this study, we consequently investigated the immune-inflammatory response in CAE patients, comparing it with both normal and atherosclerosis coronary artery response. This may provide a better understanding of CAE pathogenesis.

## 2. Methods

### 2.1. Patient Selection

We reviewed over 18,000 angiograms performed between 2003 and 2011 at the Heart Centre of the Umeå University Hospital, Sweden, and Letterkenny University Hospital, Ireland, for finding patients with clear evidence of CAE, using the conventional definition of a coronary diameter ≥ 1.5 times the diameter of the original caliber of the artery or the adjacent segment diameter, and which is not localized (>20 mm long and/or includes more than one third of the arterial length) [23]. All patients underwent coronary angiography to investigate chest pain with a positive non-invasive test. Two independent cardiologists reviewed all identified angiograms to confirm the diagnosis of CAE.

Of these angiograms, 66 patients fulfilled the defined criteria for CAE (prevalence of 0.4% with 95% CI 0.3–0.5%), 15 of them (22.7%) with pure CAE criteria with no evidence for atherosclerosis (smooth arteries). However, the remaining 51 patients had a mixed CAE with evidence for only minimal atherosclerosis changes, <20% of the lumen diameter [1], and no patient had obstructive disease (>50% luminal stenosis). A formal invitation was sent to all patients and 16 agreed to participate in this study having given formal written consent.

Cardiovascular (CV) risk factors for atherosclerotic coronary artery disease were identified from patients’ hospital records at the time of presentation, including hypertension, diabetes mellitus, smoking or ex-smoking, family history of coronary heart disease, and hyperlipidemia. Both centers used the standard definitions for risk factors according to the guidelines. Hypertension was defined according to the JNC-7 guidelines as systolic blood pressure ≥140 mmHg, diastolic blood pressure ≥90 mmHg, and/or the use of antihypertensive medications. Diabetes was defined as overnight fasting blood glucose ≥7 mmol/L (126 mg/dL), postprandial blood glucose ≥11 mmol/L (200 mg/dL), or the use of insulin or oral hypoglycemic agents. Body mass index (BMI) was calculated using height and weight measurements. Blood lipids were measured using standard enzymatic methods, and hypercholesterolemia was defined as total cholesterol ≥6.2 mmol/L (240 mg/dL), low-density lipoprotein cholesterol ≥4.14 mmol/L (160 mg/dL), or the use of lipid-lowering medications. However, all patients were on statins at the time of blood sample collection. Family history of premature CAD was noted if a male first-degree relative aged <55 or a female first-degree relative aged <65 developed CAD. Patients were classified as smokers if they had smoked during the last six months [5,24]. No CAE patient had documented inflammatory pathology at the time of the study, i.e., inflammation related to high atherosclerosis burden or connective tissue diseases, (Ehlers–Danlos syndrome, Kawasaki disease, and polycystic kidney disease), iatrogenic coronary aneurysms, or Takayasu arteritis, and this may have suggested a causal relationship for the CAE lesion.

We also studied another group of 69 patients with evidence of mild non-obstructive (<20%) coronary artery disease and a third group of 140 controls, whose angiogram showed completely normal coronary arteries. The study was approved by the Regional Ethics Committee of Umeå (Sweden “Dnr 08-118M” on 24 September 2012) and Letterkenny University Hospital ethics committee on 22 May 2012. (North West Health Service Executive, Ireland).

### 2.2. Sample Preparation and Analysis

Two hundred twenty-five serum samples (patients and controls) were assayed in duplicate using the (Merck Sharp and Dohme Co. “MSD”) pro-inflammatory Panel I, a highly sensitive multiplex enzyme-linked immunosorbent assay (ELISA) for quantitatively measuring 10 cytokines including interferon (IFN-γ), interleukin (IL)-1β, IL-2, IL-4, IL-6, IL-8, IL-10, IL-12p70, IL-13, and tumor necrosis factor (TNF-α) from sample volume (2 × 50 μL) using an electrochemiluminescent detection method (MesoScale Discovery, Gaithersburg, MD, USA). In total, the samples were distributed in three different plates that all contained standards and a pooled serum sample. The lowest detection limit (LLOD) was calculated according to the manufacturers’ protocol and the mean value for the three plates was used for further calculation of the sample concentrations. Any value below the lowest limit of detection for the cytokine assay was replaced with zero in the statistical calculations. 

### 2.3. Statistical Analysis

All quantities of samples of CAE and controls are listed. Differences between patient demographics with CAE were assessed using Mann–Whitney *U*-tests or Chi-2 tests for initially CAE vs. atherosclerotic CAD and then for CAE vs. normal controls. The quantitative data is reported as median and interquartile values. Kruskal–Wallis *H*-test was used for comparisons of all three groups, where pairwise post-hoc comparisons were performed using Dunn’s test and the Bonferroni correction for multiple testing. Points are shown as outliers if they were larger than Q3 + 2 × IQR or smaller than Q1-2 × IQR, where Q1 and Q3 are the 25th and 75th percentiles, and IQR = Q3 − Q1. Statistical significance was defined as *p* < 0.05. Analyses were performed with IBM SPSS Statistics program for Macintosh Version 24.0 (IBM Corp., Armonk, NY, USA).

## 3. Results

### 3.1. Demographics and CV Risk Factors

The baseline demographic data and CV risk factors were examined in the three groups and are presented in Table 1. There were no significant differences between CAE and CAD patients with regard to age, gender, hypertension, hyperlipidemia, diabetes mellitus, family history of ischemic heart disease, and smoking (matching selection). However, the Biobank-based selected controls were somewhat younger and included fewer subjects with hypertension and hyperlipidemia compared to CAE patients (Table 1).

### 3.2. Immuno-Inflammatory Response in CAE vs. Controls

**Figure 1:**
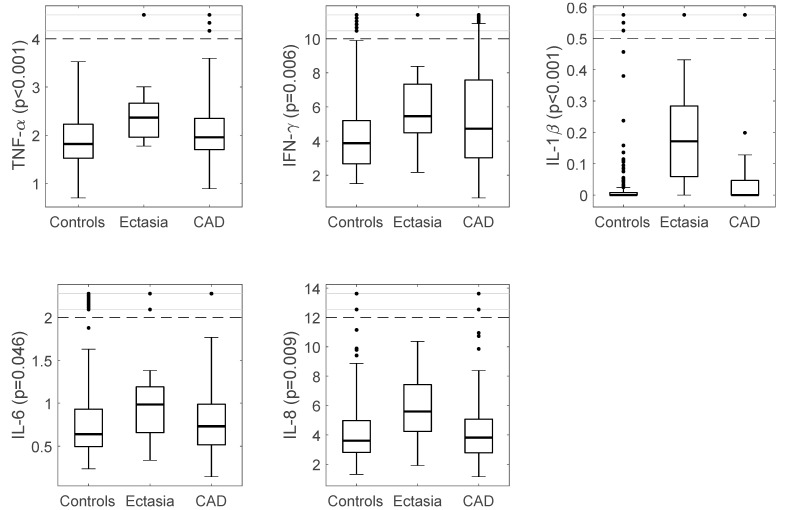
Cytokines with significantly higher levels in CAE patients compared to controls. *P*-values are derived from Kruskal–Wallis test of all three groups (see Table 2). CAE patients also presented with significantly higher levels IL-1β and IL-8 than CAD patients. Boxes show median and interquartile. Dashed lines indicate the threshold for defining extreme values, which are shown in a compressed region between the solid lines.

The 16 patients with CAE (mean age 64.9 ± 7.3 years, 6 female) were compared with the 140 matched controls (age 58.6 ± 4.1 years, 40 female). CAE patients had significantly higher systemic levels of INF-γ, TNF-α, IL-1β, and IL-8 (*p* = 0.007, 0.01, 0.001 and 0.002, respectively), while the levels of IL-2 and IL-4 were lower (*p* < 0.001 for both) than controls. The systemic levels of cytokines IL-10, IL-12P (subunits IL-12), IL-23, and IL-13 were comparable between the two groups with no measurable differences. (Table 2 and Figure 1).

### 3.3. Immuno-Inflammatory Response in CAE vs. CAD

When the CAE group was compared with the CAD group, the levels of IL-8 and IL-1β were found to be significantly higher in CAE patients (*p* = 0.023 and <0.001, respectively). However, the systemic levels of IL-2 and IL4 were lower in CAE (*p* < 0.001 for both). The levels of the other cytokines were not different between the two groups.

### 3.4. Pure Ectasia vs. Mixed Ectasia

Sub-analysis investigation of pure versus mixed ectasia was also undertaken. This showed comparable levels of all cytokines (*p* > 0.05) between pure (*n* = 6) and mixed (*n* = 10) CAE, with no significant differences in any of the examined cytokines. IL-4 was low in the mixed group (0.004 vs. 0.007 pg./L), but this was not significant (*p* = 0.06) (Table 3).

### 3.5. Data Reproducibility

All samples were run in duplicate and quantities are listed. Despite the already proven high sensitivity of the assay used, the data was reproducible with no differences between both sets of results. This confirms the accurate quantitative results.

## 4. Discussion

### 4.1. Summary of Findings

We have investigated the systemic immune-inflammatory status in a group of CAE patients, and the results show a different cytokine milieu compared to the healthy controls. CAE patients had raised systemic levels of INF-γ, TNF-α, IL-1β, IL-6, and IL-8 and lower IL-2 and IL-4 than controls. A similar pattern was also found when CAE systemic levels were compared with the CAD group. These findings likely reflect a state of increased inflammatory response through macrophage activation with resulting cytokine release. Leukocytes, and their differential subgroups, as a key player in the inflammatory process, were higher in pure CAE (Table S1) [25,26], whereas cytokines levels were comparable between patients with pure and mixed CAE.

### 4.2. Immuno-Inflammatory Response in CAE vs. Atherosclerosis

Previous studies of the immuno-inflammatory response have demonstrated [27] higher levels of IL-6 and lower IL-2 in CAE patients compared with those patients with normal and obstructive coronary disease. We encountered the same results in our study. These differences in pro-inflammatory levels suggested T helper type 2 cell (TH2) immune response involvement in CAE [27], similar to what has previously been proposed by Adioglu et al. [28]. It is well established that TNF-α plays a key role in the atherosclerosis process through stimulation of the TH1 pathway, thus leading to macrophage activation [29] (Figure 1). CAE may share the same pathogenesis of the underlying macrophage activation with higher TNF-α levels as noted in our cohort.

The higher levels of cytokines in CAE, particularly IL-6, IL-8, IFN-γ, IL-1β, and TNF-α are similar to those in atherosclerosis, where TH1 enhances macrophage activation through IFN-γ and IL-2. The detected lower level of IL-2 in CAE may suggest attenuated positive feedback of macrophage activation different from atherosclerosis, i.e., attenuated TH1 response, but macrophages still had an alternative trigger for direct activation [29]. Additionally, higher levels of IL-6 may also support increased TH2 cell production. This correspondingly may reinforce the notion of a non-atherosclerotic process in CAE. These results establish an interesting link with our previously published lipidomic profiling data, which showed lower sphingomyelins (SM) levels in CAE patients compared to controls. Low SM levels reduce lipoprotein aggregation engulfed by activated macrophages, resulting in fewer macrophage foam cell, the main component in atheroma, and hence less atheroma formation [5,29].

### 4.3. The Role of IL-6 in CAE

The raised IL-6 level in CAE is somewhat controversial, since its role in atherosclerosis appears equivocal [30,31]. IL-6 can be regarded as a pro-inflammatory cytokine but may have reduced activity through inhibiting macrophage scavenger receptor-A and hence reduced macrophage activation [32]. Likewise, IL-6 has been shown not to correlate with an increased risk of atherosclerotic coronary disease [33]. These findings suggest lower pro-inflammatory response and fewer activated macrophages, which is enough to dispute the atherosclerotic nature. However, in CAE, the levels of IL-6 were found to be higher than atherosclerosis, suggesting a pro-inflammatory response secreted by macrophages and T-cells. This would lead to T- and B-cell activation, but importantly smooth cell proliferation activation, and may enhance vascular remodeling in CAE.

### 4.4. Pro Inflammatory Pathophysiology and Macrophages Heterogeneity in CAE

The heterogeneous macrophage activation, which produces various cytokines, presents a complex interaction [16,34]. The main three populations of activated macrophages (M1–M3) are classical macrophages, wound-healing macrophages, and regulatory macrophages [35]. Conventionally, activated macrophages are those produced during cell-mediated immune response, with a predominant TH1 cell implication, as is the case in atherosclerosis. TNF-α and IFN-γ enhance macrophage activation, leading to high levels of pro-inflammatory cytokines and mediators, and hence, can cause extensive damage to the host leading to atherosclerosis and thrombosis [27,36]. The immune inflammatory response in CAE yield raised IL-1β, IL-6, TNF-α, and IFN-γ. Classic “tissue” macrophage activation, in response to innate stimuli, might occur in response to a trigger of stress or viral infections in CAE patients in addition to IFN-γ. This trigger may play a role in compensating for attenuated IL-2 levels secreted by TH1. However, the main trigger for the pro-inflammatory status remains unidentified.

### 4.5. IL-4/TH2 Role in CAE

T helper cells (TH2) secrete IL-4 and help antibody production by B-cells and wound healing macrophage activation (M2) [35]. TH2 lineage is not identified within the atherosclerotic lesions and IL-4, its prototype-related cytokine, does not significantly influence the development or regression of atherosclerotic lesions [20]. These findings partially dispute the atherosclerosis process in CAE with significantly lower IL-4 when compared to atherosclerosis as shown in our study.

### 4.6. Perturbed IL-2 Systemic Levels in CAE

Activated T-cells secrete IL-2, and their higher count are correlated with acute coronary syndrome (ACS) [37,38]. However, soluble IL-2 receptors and IL-2 systemic levels in stable CAD were found to favor the overproduction of IFN-γ [39,40]. In our study, we found lower levels of IL-2 in the CAE group when compared to atherosclerosis. This may suggest a perturbed TH1 pathway.

### 4.7. The Immuno-Inflammatory Role of CAE (TNF-α and IFN-γ) on Vascular Remodeling

**Figure 2:**
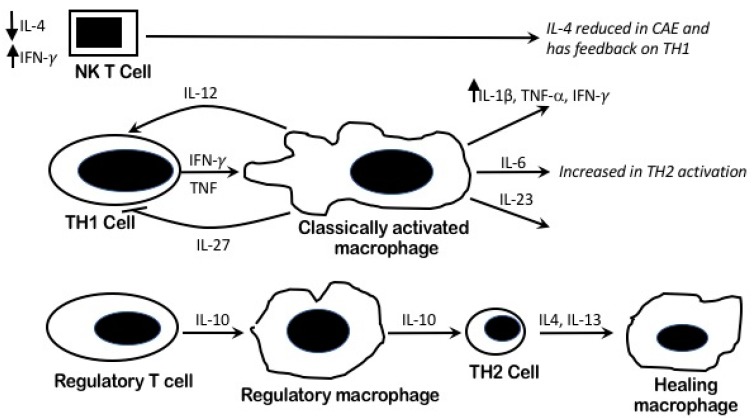
Systematic diagram to illustrate the enhanced pro inflammatory systemic response in CAE. Macrophage activation and the cytokines response. The proposed TH2 pathway activation leads to increased IL-6. However, low IL-4 levels as an outcome of possibly perturbed NK T-cell function lead to poor healing.

The raised cytokine markers in our CAE patients may reflect an exaggerated response to the enzymatic degradation of the extracellular matrix of the media producing extensive vascular remodeling [41,42]. This mechanism has been histologically confirmed as demonstrating extensive destruction of the musculo-elastic element, with marked degradation of the medial collagen and with elastin fibers with disruption of the internal and external elastic lamina [43,44]. The likely explanation for this is that the raised immune inflammatory markers of TNF-α and IFN-γ in our CAE patients induce a broad range of matrix metalloproteinases (MMPs) and inhibit collagen synthesis. These effects are mediated by leukocyte transmigration and local activation by cytokines into activated macrophages. Cell-surface adhesion molecules have been found to be elevated in pure CAE patients [35,45]. Combining this with the reduced IL-4 (i.e., reduced adaptive immunity via mast cells and TH2 response, which reduces wound healing macrophages that promote unopposed tissue damage) [46] might explain the heightened destructive vascular remodeling with CAE (Figure 2)

### 4.8. Pure vs. Mixed CAE Cytokines Profiling

The current study has shown no difference in the patterns of immune-inflammatory systemic response between pure and mixed ectasia (with very minimal atherosclerosis). However, the only weak observation was the slightly higher IL4 levels in pure CAE, but this was not supported statistically (*p* = 0.06).

### 4.9. Study Limitations

The most important limitation in this study is the small sample volume because of the rarity of the condition as well as the geographic difficulty in obtaining blood samples from all the original 66 identified CAE individuals. Patients were recruited from two different geographic locations in Sweden and Ireland; however, the cohort was homogeneous of both races. We were unable to comment on the duration of risk factors or their severity. The control group was slightly younger with fewer subjects with hypertension and hyperlipidemia when compared with CAE. On the other hand, there were no differences in demographics between CAE and CAD patients.

### 4.10. Methodological Strength

Despite the above limitations, the strength of our findings is significant. While different methodologies for biochemical analysis are available, the advantage of our multispot ELISA allowed uniform quantification of all markers at the same time, unlike other methods that might require many samples, different sample preparations and apparatus calibrations. Furthermore, our results were reproducible in the lab by the current kits when a duplicate run of the samples was performed.

## 5. Conclusions

Enhanced systemic pro-inflammatory response in CAE explains our study findings. The profile of CAE immune-inflammatory response might indicate activation of macrophages consequent to a possible viral or stress trigger. CAE cytokine milieu differs from atherosclerosis, indicating a different pathophysiology despite common conclusions of a chronic but exaggerated inflammation process. Further studies might be warranted to validate these findings.

## Figures and Tables

**Table 1:** Demographic characteristics and cardiovascular risk factors of coronary artery ectasia (CAE) group and controls.

	CAE Patients (*n* = 16)	Controls (*n* = 140)	*p*-Value CAE vs. Control	CAD Patients (*n* = 69)	*p*-Value CAE vs. CAD
Gender (Female, %)	6 (38%)	81 (58%)	NS	41 (59%)	NS
Age (mean ± SD) years	64.9 ± 7.3	58.6 ± 4.1	<0.011	64.5 ± 8.7	NS
Hypertension (N, %)	9 (56.2%)	34 (25%)	<0.01	49 (71%)	NS
Diabetes Mellitus (N, %)	4 (25%)	22 (15.7%)	NS	11 (15.9%)	NS
Hyperlipidaemia (N, %)	9 (56%)	18 (25%)	<0.001	51 (74%)	NS
BMI (mean ± SD)	25.8 ± 4.5	27 ± 4.4	NS	27.2 ± 5.6	NS
Family history of IHD (N, %)	7 (43%)	57 (40.7%)	NS	48 (69.6%)	NS
Smoking (N, %)	7 (43%)	47 (33.8%)	NS	40 (58%)	NS

NS: not significant.

**Table 2:** Immuno-inflammatory response and related cytokines levels in the coronary artery ectasia (CAE) group versus control group and coronary artery disease (CAD) patients. Data is presented as median (interquartile range) and *p*-values is derived from Kruskal–Wallis *H*-tests, using Dunn’s test with Bonferroni correction for post-hoc tests.

Cytokine	Controls (*n* = 140)	CAE Patients (*n* = 16)	*p*-Value CAE vs. Control	CAD (*n* = 69)	*p*-Value CAD vs. CAE	*p*-Value Kruskal-Wallis
**I-Equal Levels (median and interquartile)**						
IL-10 (pg./mL)	0.25 (0.15)	0.26 (0.11)	-	0.29 (0.21)	-	0.07
IL-12p (sub-unit) IL-12 and IL-23 (pg./mL)	0.09 (0.11)	0.08 (0.08)	-	0.09 (0.09)	-	0.41
IL-13 (pg./mL)	0.26 (0.60)	0.14 (0.44)	-	0.28 (0.66)	-	0.46
**II-Reduced levels (median and interquartile)**						
IL-2 (pg./mL)	0.25 (0.08)	0.12 (0.05)	<0.001	0.26 (0.11)	<0.001	<0.001
IL-4 (pg./mL)	0.04 (0.03)	0.004 (0.006)	<0.001	0.04 (0.03)	<0.001	<0.001
**III-Increased levels (median and interquartile)**						
IL-6 (pg./mL)	0.64 (0.44)	0.98 (0.60)	0.049	0.73 (0.48)	0.25	0.046
IL-8 (pg./mL)	3.62 (2.18)	5.59 (3.65)	0.007	3.82 (2.30)	0.023	0.009
IFN-γ (pg./mL)	3.88 (2.55)	5.45 (3.33)	0.032	4.72 (4.58)	0.74	0.006
IL-1β (pg./mL)	0.001 (0.01)	0.17 (0.24)	<0.001	0.00 (0.05)	<0.001	<0.001
TNF-α (pg./mL)	1.82 (0.71)	2.37 (0.74)	0.002	1.96 (0.66)	<0.12	<0.001

**Table 3:** Immuno-inflammatory response and related cytokines levels in CAE sub-groups of pure versus mixed ectasia. Data is presented as median (interquartile range) and *p*-values derived from Mann–Whitney *U*-tests.

Cytokines	Mixed Ectasia (*n* = 10)	Pure Ectasia (*n* = 6)	*p*-Value
IL-10 (pg./mL)	0.27 (0.12)	0.25 (0.19)	0.79
IL-12p (sub-unit IL-12) and IL-23 (pg./mL)	0.07 (0.07)	0.11 (0.12)	0.64
IL-13 (pg./mL)	0.18 (0.57)	0.14 (0.37)	0.71
IL-2 (pg./mL)	0.15 (0.07)	0.12 (0.03)	0.15
IL-4 (pg./mL)	0.004 (0.004)	0.007 (0.012)	0.06
IL-6 (pg./mL)	0.99 (1.06)	0.94 (0.51)	0.49
IL-8 (pg./mL)	5.67 (3.49)	4.79 (3.67)	0.37
IFN-γ (pg./mL)	5.39 (3.10)	5.96 (6.25)	0.26
IL-1β (pg./mL)	0.22 (0.37)	0.13 (0.17)	0.49
TNF-α (pg./mL)	2.37 (0.73)	2.22 (0.97)	0.49

## Supplementary Materials

The following are available online at www.mdpi.com/1422-0067/19/1/260/s1.
