# Supplementary material for: Cytokine Disturbances in Coronary Artery Ectasia Do Not Support Atherosclerosis Pathogenesis
Source: Int J Mol Sci. 2018 Jan 16;19(1):260. doi: 10.3390/ijms19010260 (PMC5796206; doi:10.3390/ijms19010260)
Supplement: Supplementary file 1 [file ijms-19-00260-s001.pdf]

Supplementary Table S1. This table helps to understand the origin, target and function of each cytokines in the cohort. Th1 = T helper 1 cells, Th2 = T helper 2 cells. SMC = smooth muscles cells, NK = natural killer cells, EC = endothelial cells, IFN = interferon.

Cytokine	Cellular origin	Cell intention	Cellular properties and actions
I-Equal Levels (median and interquartile)			
IL-10 (pg./ml)	Macrophages, Th2, B cells, mast cells	Macrophages, T & B cells	Anti inflammatory, inhibits Th1, Promotes proliferation and differentiation of regulatory T cels
IL-12p (sub-unit) IL-12 and IL-23 (pg./ml)	Th1 cells	T cells, macrophages	Proinflammatory; promotes NK and cytotoxic lymphocyte activity; induces IFN- γ
IL-13 (pg./ml)	Th2 cells	B cells	Activation of Ig transcription
II- Reduced levels (median and interquartile)			
IL-2 (pg./ml)	Activated T cells	Macrophages, T & B cells, NK cells	T-cell growth factor, stimulates NK activity, stimulates Treg cells
IL-4 (pg./ml)	Th2 cells, mast cells	T & B cells, mast cells, macrophages, hematopoietic progenitors	Proliferation and differentiation of B cells (Ig switching to IgG1 and IgE) and Th2 cells (anti-inflammatory by inhibiting Th1 immune responses); stimulates VCAM-1
III- Increased levels (median and interquartile)			
IL-6 (pg./ml)	Macrophages, EC, SMC, T cells	T & B cells, hepatocytes, EC, SMC	Differentiation of myeloid cells, induction of acute phase proteins, SMC proliferation
IL-8 (pg./ml)	Monocytes, EC, T cells	Neutrophils, T cells, monocytes	Proinflammatory, promotes leukocyte arrest
IFN-γ (pg./ml)	Th1 cells, NK cells, SMC	Macrophages, lymphocytes, NK cells, EC, SMC	Proinflammatory, promotes Th1 immune responses/secretion of Th1-associated cytokines, inhibits extracellular matrix synthesis by SMC
IL-1β (pg./ml)	Activated T cells	Macrophages, T & B cells, NK cells	T-cell growth factor, stimulates NK activity, stimulates Treg cells
TNF-α (pg./ml)	Macrophages, T & B cells, NK cells, SMC	Many cell types	Proinflammatory, fever, neutrophil activation, bone resorption, anticoagulant, tumour necrosis
